# A fast‐screening approach for the tentative identification of drug‐related metabolites from three non‐steroidal anti‐inflammatory drugs in hydroponically grown edible plants by HPLC‐drift‐tube‐ion‐mobility quadrupole time‐of‐flight mass spectrometry

**DOI:** 10.1002/elps.202000292

**Published:** 2020-12-21

**Authors:** Franz Mlynek, Markus Himmelsbach, Wolfgang Buchberger, Christian W. Klampfl

**Affiliations:** ^1^ Institute of Analytical Chemistry Johannes Kepler University Linz Austria

**Keywords:** Drift‐tube ion mobility‐mass spectrometry, Environmental analysis, Fast‐screening, NSAIDs, Pharmaceuticals, Plant metabolism

## Abstract

The (tentative) identification of unknown drug‐related phase II metabolites in plants upon drug uptake remains a challenging task despite improved analytical instrument performance. To broaden the knowledge of possible drug metabolization, a fast‐screening approach for the tentative identification of drug‐related phase II metabolites is presented in this work. Therefore, an *in silico* database for the three non‐steroidal anti‐inflammatory drugs (ketoprofen, mefenamic acid, and naproxen) and a sub‐group of their theoretical phase II metabolites (based on combinations with glucose, glucuronic acid, and malonic acid) was created. Next, the theoretical exact masses (protonated species and ammonia adducts) were calculated and used as precursor ions in an autoMS/MS measurement method. The applicability of this workflow was tested on the example of eleven edible plants, which were hydroponically grown in solutions containing the respective drug at a concentration level of 20 mg/L. For the three drugs investigated this led to the tentative identification of 41 metabolites (some of them so far not described in this context), such as combinations of hydroxylated mefenamic acid with up to four glucose units or hydroxylated mefenamic acid with two glucose and three malonic acid units.

**Abbreviations**
AJS‐ESI: Agilent jet stream electrospray ionization
DCF: diclofenac
DT‐IM‐QTOF: drift tube ion mobility Q‐TOF
Glc: glucose
KPF: ketoprofen
Mal: malonic acid
MeOH: methanol
MFA: mefenamic acid
NPX: naproxen
NSAIDs: non‐steroidal anti‐inflammatory drugs
WWTPs: wastewater treatment plants

## 1 Introduction

The problem of water scarcity and droughts in Europe was first addressed by a Communication of the European Commission (COM (2007) 414 final) in 2007 [1]. Therein it was mentioned that irrigation in agriculture, amongst others, might have a significant effect on water resources in the future. Crop irrigation, which so far was just a common practice in arid regions [2], will become more important in many regions in Europe to ensure a steady supply of foodstuff. Droughts also affect the groundwater level resulting in a reduction of the amount of drinking water available [3]. For this reason, groundwater cannot be regarded as a reliable water source for irrigation in agriculture in the future. A promising alternative for irrigation purposes is the usage of treated wastewater. Unfortunately, even after treatment in waste‐water treatment plants (WWTPs), wastewater may contain unwanted contaminants. Amongst other groups of substances, detectable amounts of various pharmaceuticals have been found in the effluents of WWTPs [4, 5, 6, 7, 8, 9, 10]. In a study commissioned by the Austrian Federal Ministry for Agriculture, Regions and Tourisms the presence of 85 pharmaceuticals in 10 Austrian surface waters (rivers) and 10 wastewaters was investigated [11]. Thereby particularly the most widely applied types of pharmaceuticals were detected. An important group in this context are non‐steroidal anti‐inflammatory drugs (NSAIDs). Next to the most widely used NSAID diclofenac, also the three analgesics mefenamic acid (MFA), naproxen (NPX) and ketoprofen (KPF) were found above the limit of detection in most of the analyzed water samples.

If reclaimed waters are used for the irrigation of crops, contaminants (e.g., drugs) might come into contact with (edible) plants and may be taken up by the plant and/or probably even further metabolized. In the case of edible plants, such potentially harmful substances may end up in the food chain. As more and more countries rely on the use of reclaimed waters in agriculture, monitoring plants used for the production of food and feed regarding the presence of some of the most widely prescribed drugs and their major metabolites might be beneficial. This fact has already been the topic of a series of studies dealing with the uptake and/or the further metabolization of pharmaceuticals (and personal care products) in a series of plants (for reviews see [12, 13, 14, 15, 16, 17]).

Regarding NSAIDs, most studies published so far were focused on diclofenac. Only a few papers exist that deal with the fate of other important representatives of the NSAID‐family, such as MFA, NPX and KPF, upon interaction with plants [18, 19]. In the present paper we report an approach based on HPLC coupled to high‐resolution tandem mass spectrometry (HR‐MS/MS) for the fast screening of plant extracts for the presence of parent drugs and their metabolites. For this study we used MFA, NPX and KPF as test substances, investigating their interaction with a range of edible plants (salad, carrots, pepper, radish, chive, onions, pea, tomato, maize, sorghum, and amaranth) grown hydroponically. Hydroponic conditions have been chosen, as they allow working with exactly defined amounts of the parent drug and avoid lengthy growing times as encountered in experiments conducted in real soil. Nevertheless it can be assumed, that the proposed methodology will be suitable for the soil‐grown plantlets as well. For this reason, in a first step an *in silico* database was created, including the three parent drugs, their potential phase I metabolites and tentative phase II metabolites formed from these molecules by interaction with a series of biologically important substances such as sugars, amino acids or small organic acids. Subsequently, this database was employed for the analysis of extracts from plants treated with the three pharmaceuticals.

## 2 Materials and methods

### 2.1 Reagents and samples

The pharmaceutical formulations of ketoprofen (Profenid 200 mg, Sanofi) and naproxen (Naprobene 500mg, Ratiopharm) were bought in a local pharmacy. Mefenamic acid and formic acid ACS (≥ 96%) were purchased from Sigma‐Aldrich (Steinheim, Germany). For structural formulas of the drugs see Figure S1. Hydrochloric acid (37%), acetonitrile (ACN) and methanol (MeOH) were supplied by VWR Chemicals (Vienna, Austria). Ultrapure water was obtained from a Milli‐Q water purification system (Millipore, Bedford, MA, USA).

10000 mg/L stock solutions of the respective NSAID were prepared in MeOH and further diluted in tap water for plant irrigation. The irrigation water contained a maximum amount of 200 μL MeOH in 100 mL tap water (20 mg drug/L) in the growing experiments. It was necessary to keep the MeOH concentration low to avoid further stress for the plants.

Lettuce (Maikönig), carrots (Rote Riesen 2), pepper (Neusiedler Ideal), radish (Saxa 3), chive (Nelly) and onions (Weiße Königin) were all from Kiepenkerl (Everswinkel, Germany) and purchased in a local garden shop. Pea (*Pisum sativum*, cv. Premium) and tomato (*Solanum lycopersicum*, cv. Bajaja) were purchased from MoravoSeed CZ Corporation (Czech Republic) and maize (*Zea mays*, cv. Agnan) from Oseva Agro Brno Ltd. (Czech Republic). Amaranth was obtained from dm‐drogeriemarkt GmbH (Karlsruhe, Germany) and sorghum from Raiffeisen Ware Austria AG (Korneuburg, Austria).

### 2.2 Instrumentation

A 1260 Infinity II HPLC system was coupled with an Agilent Technologies 6560 Drift Tube Ion Mobility Q‐TOF (DT‐IM‐QTOF), which was equipped with a Dual Agilent Jet Stream Electrospray Ionization (Dual AJS‐ESI) source and a gas kit (Alternate Gas Kit, Agilent Technologies). The separation column was an Agilent InfinityLab Poroshell 120 Bonus‐RP column (100 × 3.0 mm, 2.7 μm) combined with a C18 Guard column (4 × 3.0 mm, Phenomenex). The Dual AJS‐ESI source was operated in positive mode. Nitrogen was used as drying gas and sheath gas, operated with a flow rate of 10 L/min and the gas temperatures were 275°C. The injection volume was set to 25 μL and the nebulizer gas pressure was 50 psi. The following voltages were applied: capillary (3500 V), nozzle (1000 V) and fragmentor (400 V). The DT‐IM‐QTOF was auto‐tuned in the “2 GHz extended dynamic range” mode in the “750 m/z fragile ions” mode.

The mobile phase was **A** ultrapure water with 0.1% formic acid (v/v) and **B** acetonitrile with 0.1% formic acid (v/v).

The following HPLC gradient elution was employed for samples containing MFA: 20% B from 0 min until 0.5 min, 95% B until 11 min, hold 95% B for 2 min, re‐conditioning with 20% B for 5 min.

For samples containing KPF and NPX, the following HPLC gradient elution was used: 20% B from 0 min until 0.5 min, 55% B until 10.5 min, 95% B until 13 min, hold 95% B for 2 min, re‐conditioning with 20% B for 5 min.

Acquisition parameters for autoMS/MS(Seg): QTOF‐only acquisition mode, acquisition rate MS (1 spectra/s), acquisition rate MS/MS (0.6 spectra/s), isolation width (narrow ∼ 1.3 amu), fixed collision energy (5 V), precursor abs. threshold (15000 counts), active exclusion list (enabled) – excluded after 2 spectra, use preferred ion list only (checked).

Acquisition parameters for collision cross section (^DT^CCS_N2_) determination: IM‐QTOF acquisition mode, 4‐bit multiplexing, IM trap fill time (3900 μs), trap release time (250 μs), frame rate (0.9 frames/s), IM transient rate (18 transients/frame), maximum drift time (60 ms). For ^DT^CCS_N2_ determination, a single field calibration had to be done with the Agilent Tune Mix prior to the sample measurement. Based on suggestions by Stow et al. [20] advanced parameters were as follows: Drift Tube Entrance (1567 V), Drift Tube Exit (217 V), Rear Funnel Entrance (210.5 V), Rear Exit Funnel (38 V). These four parameters were fixed in the acquisition method independent of the tune parameters.

### 2.3 Germination of seeds and growing of plantlets

The bottom of a Petri dish was covered with filter paper, which was soaked with drug‐containing water (20 mg/L KPF, MFA or NPX). Thereon the seeds of lettuce, tomato, onion, pepper, amaranth, carrot, radish, and chive were spread and germinated in the dark for 7 days.

The cultivation of maize, pea and sorghum was as follows: soaking of the seeds in tap water overnight, germination on wetted kitchen roll for 2 days in the dark, transfer of the germinated seeds into dishes filled with ironing beads and germination for additional 5 days under a growing lamp with 16/8 hours day/night rhythm. Then the plantlets were transferred into Erlenmeyer flasks filled with drug‐containing water (20 mg/L KPF, MFA or NPX) and grown for 7 days.

### 2.4 Harvesting, extraction and analysis of plantlets and germinated seeds

The germinated seeds grown in Petri dishes and the plantlets grown in Erlenmeyer flasks were washed twice with tap water and dabbed dry by a kitchen roll. The plantlets were further separated into a root, shoot and stem part. About 1.5 g of plant material (wet weight) was weighed into a 15 mL centrifugation tube, 2 mL of 0.1 M HCl and 1 mL of ACN were added and the samples were homogenized for 10 minutes using a Star‐Beater Ballmill (VWR International, Vienna, Austria) at a frequency of 18 Hz. The samples were centrifuged for 8 minutes at 4500 rpm, filtered through 0.45 μm Rotilab syringe filters into HPLC glass vials and stored at –80°C until analysis.

### 2.5 Data evaluation

For data evaluation, Agilent MassHunter Qualitative Analysis B.07.00, MassHunter PCDL Manager B.08.00, PNNL PreProcessor (2020.03.23), and IM‐MS Browser B.10.00 were used.

IM data files were first demultiplexed using PNNL PreProcessor using the following settings: demultiplexing (checked), moving average smoothing (checked) – m/z (not used) – drift (3) – chromatography/infusion (not used), remove spikes (checked), saturation repair (checked) – repair points above abundance limit: 40%. The demultiplexed files were then calibrated with the recorded single field tune using IM‐MS browser. ^DT^CCS_N2_ values were determined using feature extraction (IMFE) in the IM‐MS Brower with the following settings: Chromatographic processing of “common organic molecules” with a limited charge state of z < = 1–2. The Ion intensity was set to > = 300 and the retention time was restricted to 1–15 min.

## 3 Results and discussion

### 3.1 Design of a fast‐screening approach for detecting unknown drug‐related metabolites from plant extracts employing a lab‐made database

Starting point was knowledge acquired in our group from prior experiments focused on the uptake and metabolization of four NSAIDs (diclofenac (DCF), MFA, KPF, NPX) by garden cress (*lepidum sativum)* [18, 19]. From that research, it became obvious that besides phase I metabolites also phase II metabolites of DCF were formed mainly by conjugation with glucose (Glc) and malonic acid (Mal). Thereby compounds like DCF‐Glc‐Glc‐Mal or DCF‐Glc‐Glc‐Mal‐Mal could be detected in the extracts from plants treated with DCF, an observation that could be verified later on the example of a variety of other edible plants [21]. To facilitate the detection of this type of drug‐metabolites in plants grown hydroponically in drug‐containing waters, an *in silico* database including a large number of potential metabolites (all based on conjugation with glucose, malonic acid, and/or glucuronic acid) was created. Assuming that phase II conjugation occurs for example by addition of one or more (for practical reasons the maximum number was restricted to six of each kind) glucose or malonic acid moieties these were combined in every possible way with either the parent drug or its related phase I metabolites. Additionally, the presence of the less frequently found glucuronic acid moieties (metabolites formed by glucuronidation are primarily known from animal studies) was also considered, as glucuronidation was previously detected in maize and amaranth [21]. For this purpose, the theoretical exact masses for the potential metabolites were calculated (as H^+^ and NH_4_^+^ adducts) and used as precursor ions in a preferred ion list. Following this procedure, a so called autoMS/MS(Seg) method was generated. The latter refers to a data‐dependent acquisition mode, triggering an MS/MS event with a certain collision energy (CE) when the threshold of 15000 counts for an ion of the preferred ion list is surpassed. Thereby an appropriate CE protocol has to be chosen that is strong enough to cause fragmentation of the compound isolated by the quadrupole, but still soft enough to maintain the signal for the molecular ion in the mass spectra. Preliminary tests using CE values of 20, 15, 10 and 5 V revealed that a value of 5 V complied with these pre‐requisites. Recorded files were then analyzed using the Agilent Qual Browser, resulting in a list with extracted MS/MS spectra at a given retention time, but no compound name. This list of possible hits was introduced into the lab‐made *in silico* database, assigning the corresponding compound names. The final step was to check the MS/MS‐spectra for the appearance of the related parent drug or phase I metabolite and their characteristic fragments. Additionally, a search for fragments showing neutral mass losses, like 162.05 Da for a glucose moiety or 176.03 Da for glucuronic acid was executed. This workflow can be regarded as a “fast‐screening” approach, because in only one run MS and MS/MS information can be obtained that leads to a fast tentative identification of so far unknown drug‐related substances formed within a plant.

As a drift‐tube ion‐mobility quadrupole time‐of‐flight / mass spectrometer (DT‐IM QTOF‐MS) was employed, additionally drift times were recorded allowing the calculation of ^DT^CCS_N2_. These can be used as an additional compound‐characteristic parameter like retention time and exact mass, helping to distinguish between substances in overlapping peaks, a strategy that was already employed successfully for similar analytical challenges [21, 22, 23]. To investigate the feasibility of this approach, a series of edible plants (pepper, sorghum, tomato, onion, radish, chive, maize, amaranth, pea and lettuce) were grown in water containing either MFA, KPF or NPX. After harvesting the plants were extracted and the resulting solutions analyzed by the procedure described above.

### 3.2 Examples for the tentative identification of potential metabolites by the fast‐screening approach

**Figure 1:**
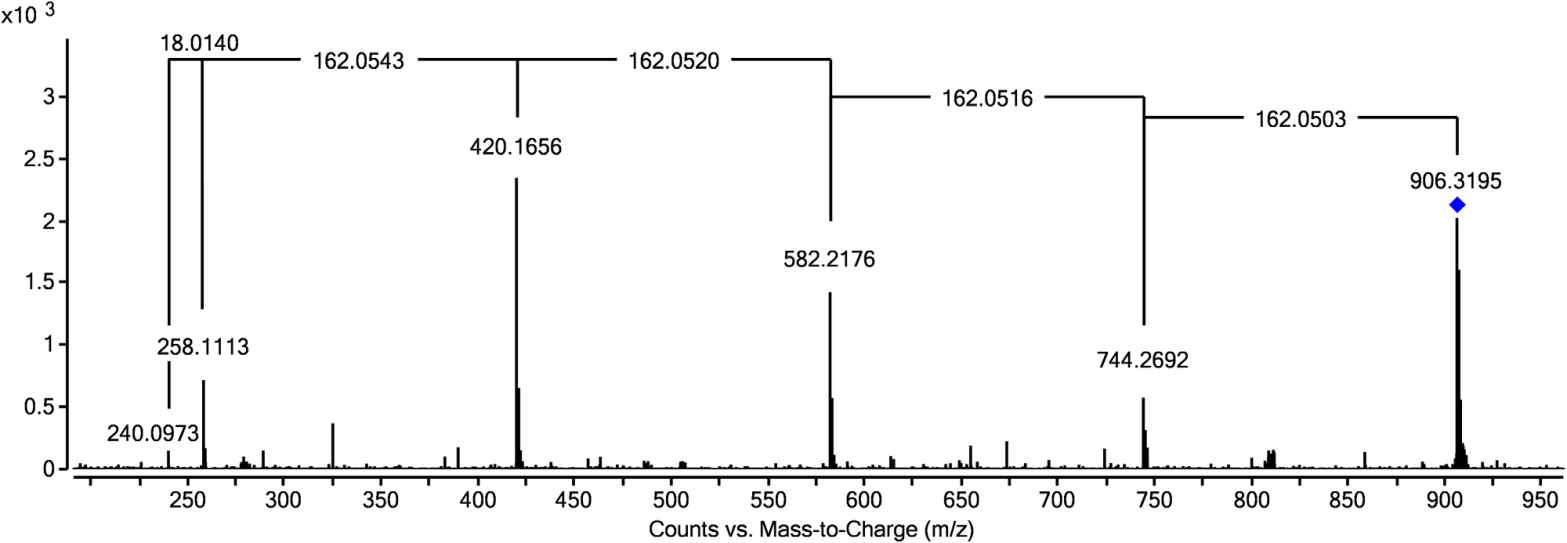
MS/MS spectra of MFA‐OH‐Glc‐Glc‐Glc‐Glc presented as protonated species and tentatively identified in tomato recorded with a CE of 5 V

Slight differences between the MS spectra of MFA‐derived substances (which were mostly detected as protonated species) and those from KPF and NPX (occurring as ammonia adducts) were observed. In previous papers NSAID‐based metabolites with up to two glucose units were described [18, 19, 21]. Employing the approach from the present study, an MFA‐OH related metabolite with four glucose moieties could be detected in extracts from tomato grown hydroponically in water containing 20 mg/L of the drug (see Figure 1). The molecular ion (906.3195 m/z) was isolated as protonated species; four fragments 744.2692 m/z, 582.2176 m/z, 420.1656 m/z, and 258.1113 m/z always showed a neutral loss of 162.05 Da, a mass difference characteristic for a glucose moiety. In addition, a fragment at 240.0973 m/z was visible in the mass spectra, which could be assigned to a water loss (−18.01 Da) from 258.1113 m/z (MFA‐OH). As a second example an interesting compound including two glucose and three malonic acid moieties attached to hydroxylated MFA (MFA‐OH‐Glc‐Glc‐Mal‐Mal‐Mal) found in chive should be mentioned (see Figure S2). From the MS/MS spectra it seems likely that two Mal are directly bound to Glc moieties, as they are cleaved off together, resulting in neutral mass losses of 248.05 Da. The third Mal seems to be bound directly to MFA‐OH, as it is present in the MS/MS spectrum as a single neutral mass loss of 86.00 Da (relating to malonic acid minus water). MS/MS spectra for two more of the drug‐related products found in plant extracts can be seen in Figure S3 (KPF‐Glc‐Glc‐Glc‐Mal) and Figure S4 (KPF‐Glc‐GlcA‐Mal) respectively.

### 3.3 Metabolites from MFA, KPF and NPX tentatively identified in various edible plants

**Figure 2:**
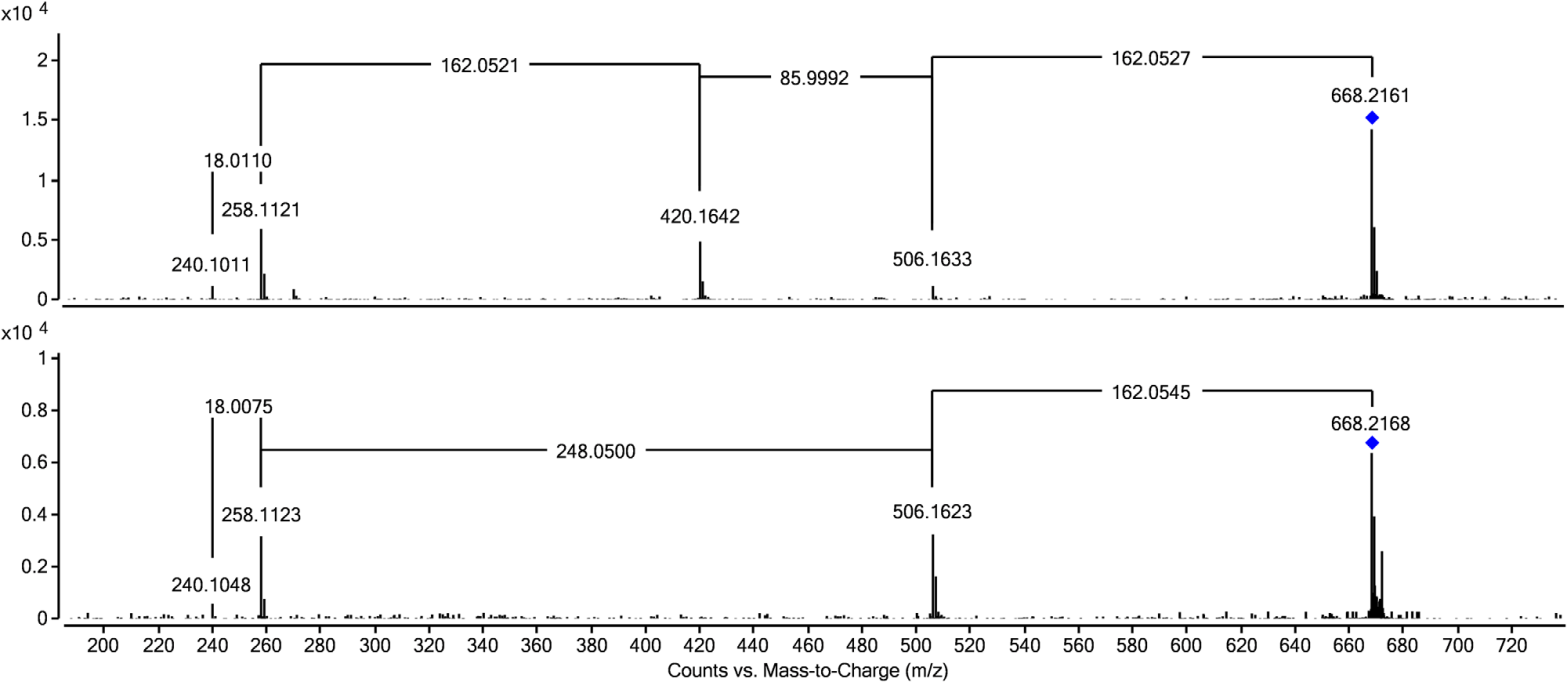
MS/MS, spectra of MFA‐OH‐Glc‐Glc‐Mal. Upper trace compound eluting at 4.2 min (^DT^CCS_N2_ value: 251.1 Å^2^); lower trace compound eluting at 5.2 minutes (^DT^CCS_N2_ value: 241.4 Å^2^)

Within the presented study, eleven different edible plants (lettuce, carrots, pepper, radish, chive, onions, pea, tomato, maize, sorghum, and amaranth) were treated with the three selected NSAIDs, harvested, and the resulting plant extracts investigated for potential drug‐related metabolites employing the approach described above. The results from this research are depicted in Tables 1, 2, 3. Thereby compounds derived from one of the three drugs conjugated with up to four glucose (MFA‐OH‐Glc_4_) and up to three malonic acid (MFA‐OH‐Glc_2_‐Mal_3_) units could be found. These compounds were all detected using our database. As can be seen from the data provided within these Tables, for a large portion of the tentatively identified metabolites several chromatographically (mostly only partially) separated signals with fractionally different ^DT^CCS_N2_ values could be detected, for identical m/z values. The value for n given in the Tables relates to the maximum of chromatographically separated peaks detected in one single plant. In many cases also for the MS/MS spectra only insignificant differences could be found. These different signals might refer to structural isomers as both the hydroxyl group for phase I metabolites, as well as the compounds further added forming the phase II metabolites, can be attached to the parent substance in different ways. One example, where two chromatographically separated peaks with same accurate mass (m/z 668.2160) show differences in the MS/MS, most probably related to structural isomers, is MFA‐OH‐Glc‐Glc‐Mal. Inspecting the MS/MS spectra (see Figure 2) reveals that for the peak at 5.2 minutes first a loss of glucose is observed. In the next step, the second glucose is lost together with malonic acid followed by a loss of water. The signal at 4.2 minutes shows distinct differences as here, after the cleavage of the first glucose, a loss of malonic acid moiety followed by losses of the second glucose again followed by water was observed. This was also reflected in different ^DT^CCS_N2_ values of 251.1 Å^2^ (4.2 min) and 241.4 Å^2^ (5.2 min).

**Table 1:** Naproxen metabolites tentatively identified in 10 edible plants. (*n* = number of found structural isomers, m/z = mean of measured values)

Name	Formula	RT [min]	m/z [NH_4_^+^]	CCS [Å^2^]	*n*	Pepper	Sorghum	Tomato	Onion	Radish	Chive	Maize	Amaranth	Pea	Salad
NPX‐Glc‐Glc‐Glc	C_32_H_44_O_18_	3.7–4.7	734.2876	254.2–259.5	2	√	X	√	X	X	X	X	X	X	X
NPX‐OH‐Glc	C_20_H_24_O_9_	4.1–5.0	426.1745	196.3–203.3	2	X	X	X	X	X	√	√	√	√	X
NPX‐Glc‐Glc	C_26_H_34_O_13_	5.1–6.1	572.2319	219.0–241.6	4	√	√	√	√	√	√	√	X	√	√
NPX‐Glc‐GlcA	C_26_H_32_O_14_	5.6	586.2116	224.8	1	X	X	X	X	X	X	X	√	X	X
NPX‐OH‐Glc‐Mal	C_23_H_26_O_12_	5.8	512.1742	211.5	2	X	X	X	X	X	X	X	√	√	X
NPX‐Glc	C_20_H_24_O_8_	6.2–6.5	410.1798	196.3–198.4	2	√	√	√	√	√	√	√	√	√	√
NPX‐Glc‐GlcA‐Mal	C_29_H_34_O_17_	7.1	672.2118	243.2	1	X	X	X	X	X	X	X	√	X	X
NPX‐Glc‐Glc‐Mal	C_29_H_36_O_16_	6.1–7.6	658.2317	234.4–246.3	4	X	X	√	√	√	√	√	√	√	√
NPX‐Glc‐Glc‐Mal‐Mal	C_32_H_38_O_19_	7.9–8.2	744.2319	249.1–250.7	2	X	X	X	√	√	√	X	X	X	√
NPX‐Glc‐Mal	C_23_H_26_O_11_	8.1–9.4	496.1799	211.4–222.4	5	X	√	√	√	√	√	√	√	√	√

**Table 2:** Ketoprofen metabolites tentatively identified in10 edible plants. (*n* = number of found structural isomers, m/z = mean of measured values)

Name	Formula	RT [min]	m/z [NH_4_^+^]	CCS [Å^2^]	*n*	Pepper	Sorghum	Tomato	Onion	Radish	Chive	Maize	Amaranth	Pea	Salad
KPF‐Glc‐Glc‐Glc	C_34_H_44_O_18_	3.9	758.2848	270.9	1	√	X	√	X	X	X	X	X	X	X
KPF‐OH‐Glc‐Glc	C_28_H_34_O_14_	5.1–6.1	612.2268	238.8–241.1	3	√	X	√	X	X	X	X	X	√	X
KPF‐OH‐Glc	C_22_H_24_O_9_	4.8	450.1739	210.3	1	X	X	√	X	√	X	√	X	√	X
KPF‐Glc‐Glc	C_28_H_34_O_13_	5.1–6.1	596.2321	226.1–241.6	3	√	X	√	√	√	√	√	X	√	√
KPF‐Glc‐Glc‐Glc‐Mal	C_37_H_46_O_21_	5.1	844.2853	273.5	1	X	X	√	X	X	X	X	X	X	X
KPF‐Glc‐GlcA	C_28_H_32_O_14_	5.8	610.2105	239.3	1	X	X	X	X	X	X	X	√	X	X
KPF‐OH‐Glc‐Mal	C_25_H_26_O_12_	7.7	536.1737	208.9	1	X	X	X	X	X	X	√	X	X	X
KPF‐Glc	C_22_H_24_O_8_	6.5	434.1803	206.4	1	√	√	√	√	√	√	√	√	√	√
KPF‐Glc‐GlcA‐Mal	C_31_H_34_O_17_	7.3	696.2105	251.6	1	X	X	X	X	X	X	X	√	X	X
KPF‐Glc‐Glc‐Mal	C_31_H_36_O_16_	6.1–7.6	682.2309	248.8–254.9	4	X	X	√	√	X	√	√	√	√	√
KPF‐Glc‐Glc‐Mal‐Mal	C_34_H_38_O_19_	8.0–8.4	768.2330	253.3–260.4	1	X	X	X	√	X	√	X	X	X	√
KPF‐Glc‐Mal	C_25_H_26_O_11_	7.8–9.5	520.1801	218.1–226.9	5	X	√	√	√	√	√	√	√	√	√

**Table 3:** Mefenamic acid metabolites tentatively identified in 10 edible plants. (*n* = number of found structural isomers, m/z = mean of measured values)

Name	Formula	RT [min]	m/z [H^+^]	CCS [Å^2^]	*n*	Pepper	Sorghum	Tomato	Onion	Radish	Chive	Amaranth	Pea	Salad	Carrot
MFA‐OH‐Glc‐Glc‐Glc‐Glc	C_39_H_55_NO_23_	1.5	906.3238	293.7	1	X	X	√	X	X	X	X	X	X	X
MFA‐OH‐Glc‐Glc‐Glc	C_33_H_45_NO_18_	1.9	744.2722	253.5–272.9	2	X	X	√	X	X	X	X	X	X	X
MFA‐OH‐Glc‐Glc	C_27_H_35_NO_13_	2.8	582.2163	225.8	1	√	X	√	X	√	X	√	X	√	X
MFA‐OH‐Glc‐Glc‐Glc‐Mal	C_36_H_47_NO_21_	3.5–3.7	830.2685	265.9–281.3	1	X	X	√	√	X	X	X	√	X	X
MFA‐OH‐Glc‐Glc‐Mal‐Mal‐Mal	C_36_H_41_NO_22_	6.7	840.2153	284.3	1	X	X	X	X	X	√	X	X	X	X
MFA‐OH‐Glc‐Glc‐GlcA‐Mal	C_36_H_45_NO_22_	3.6	844.2476	266.6	1	X	X	X	X	X	X	√	X	X	X
MFA‐OH‐Glc‐Glc‐Mal	C_30_H_37_NO_16_	4.1–6.0	668.2160	241.4–252.9	4	X	√	√	√	√	√	√	√	√	√
MFA‐Glc‐Glc‐Glc	C_33_H_45_NO_17_	4.7	728.2752	249.9	1	X	X	√	X	X	X	X	X	X	X
MFA‐OH‐Glc‐GlcA‐Mal	C_30_H_35_NO_17_	4.7–5.8	682.1957	241.3–246.2	1	X	X	X	X	X	X	√	X	X	√
MFA‐OH‐Glc‐Glc‐Mal‐Mal	C_33_H_39_NO_19_	5.0–5.5	754.2163	260.2–271.2	3	X	X	X	√	X	√	X	√	√	√
MFA‐Glc‐Glc	C_27_H_35_NO_12_	5.6	566.2219	230.0	1	√	√	√	√	√	√	X	√	X	X
MFA‐OH‐Glc	C_21_H_25_NO_8_	5.4–5.7	420.1645	192.3–205.7	2	√	√	√	√	√	√	√	√	√	√
MFA‐OH‐Glc‐Mal	C_24_H_27_NO_11_	4.1–7.4	506.1638	205.3–227.3	5	√	√	√	√	√	√	√	√	√	√
MFA‐Glc‐GlcA	C_27_H_33_NO_13_	5.6	580.2009	231.9	1	X	X	X	X	X	X	√	X	X	√
MFA‐Glc‐Glc‐Glc‐Mal	C_36_H_47_NO_20_	5.8	814.2732	275.6	1	X	X	X	√	X	√	X	X	X	X
MFA‐Glc‐Glc‐Mal	C_30_H_37_NO_15_	5.9–7.2	652.2211	235.4–253.1	5	X	√	√	√	√	√	√	√	√	√
MFA‐Glc‐GlcA‐Mal	C_30_H_35_NO_16_	6.5–7.3	666.2011	238.8–247.5	2	X	X	X	X	X	X	√	X	X	√
MFA‐Glc‐Glc‐Mal‐Mal	C_33_H_39_NO_18_	7.1	738.2212	245.4	1	X	X	X	√	X	√	X	√	X	√
MFA‐Glc‐Mal	C_24_H_27_NO_10_	6.9–8.4	490.1689	209.7–219.2	3	X	√	√	√	√	√	√	√	√	√

## 4 Concluding remarks

The presented “fast‐screening” approach led to the tentative identification of a substantial number of drug‐related metabolites (ten for NPX, 19 for MFA, and 12 for KPF) in eleven edible plants. Many of these metabolites had not been described in this context before. The most important criteria for the creation of the *in silico* database was the knowledge about possible phase I metabolites of the selected pharmaceutical. Whereas in the present work only conjugation of the parent drug or one of its phase I metabolites with glucose, glucuronic acid and malonic acid was considered, manual inspection of MS/MS spectra revealed that also other small molecules of biological relevance have to be considered as potential compounds for the formation of conjugates. Therefore, it seems reasonable to expand the database by inclusion of, e.g., amino acids in future work. Focusing on a potential application of the proposed methodology it seems reasonable that in particular countries employing reclaimed waters for agricultural use in large quantities might consider the screening of plants used for the production of food and feed with respect to the presence of pharmaceuticals and their major metabolites.

*The authors declared no conflict of interest*.

## Supporting information

Supporting information

## Data Availability

All relevant data are included in the manuscript.
