# Supplementary material for: A fast‐screening approach for the tentative identification of drug‐related metabolites from three non‐steroidal anti‐inflammatory drugs in hydroponically grown edible plants by HPLC‐drift‐tube‐ion‐mobility quadrupole time‐of‐flight mass spectrometry
Source: Electrophoresis. 2020 Dec 21;42(4):482–9. doi: 10.1002/elps.202000292 (PMC7898722; doi:10.1002/elps.202000292)
Supplement: Supplementary file 1 — Supporting information [file ELPS-42-482-s001.docx]

**SUPPLEMENTARY INFORMATION TO:**

**A fast-screening approach for the tentative identification of drug-related metabolites from three non-steroidal anti-inflammatory drugs in hydroponically grown edible plants by HPLC-drift-tube-ion-mobility quadrupole time-of-flight mass spectrometry**

Franz Mlynek*, Markus Himmelsbach, Wolfgang Buchberger, Christian W. Klampfl

Institute of Analytical Chemistry, Johannes Kepler University, Altenbergerstrasse 69, 4040 Linz, Austria

*Correspondence to: Franz Mlynek (E-mail: franz.mlynek@jku.at), Tel +43 732 2468 9702


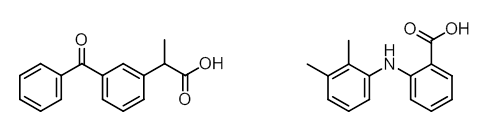


**1 2**


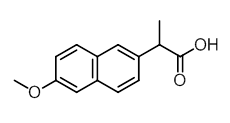


**3**

Figure S1: Structural formulas for ketoprofen (1), mefenamic acid (2) and naproxen (3).


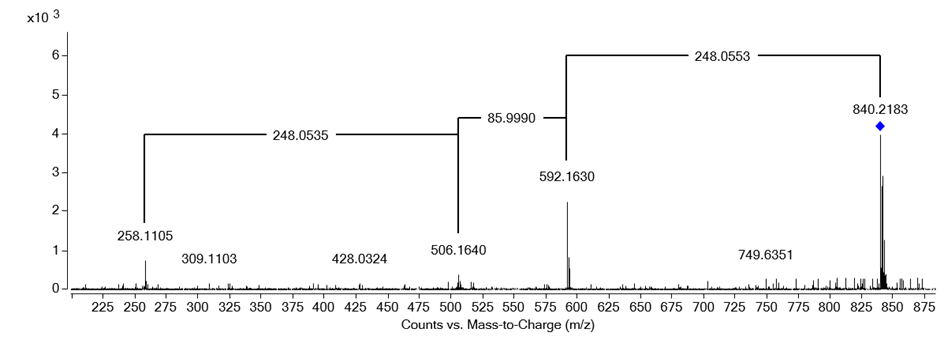


Figure S2: MFA-OH-Glc-Glc-Mal-Mal-Mal present as protonated species tentatively identified in chive recorded with a CE of 5 V.


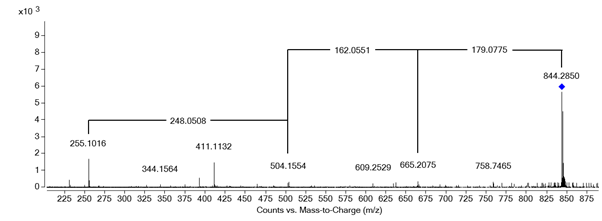


Figure S3: KPF-Glc-Glc-Glc-Mal present as ammonia adduct tentatively identified in tomato recorded with a CE of 5 V.


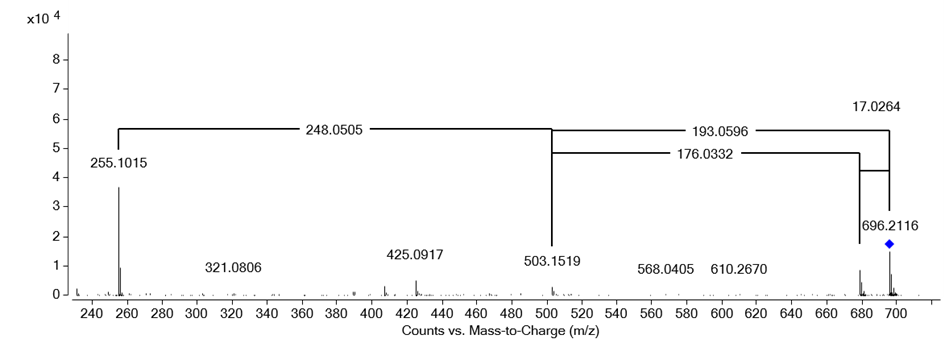


Figure S4: KPF-Glc-GlcA-Mal present as ammonia adduct tentatively identified in amaranth recorded with a CE of 5 V.
